# Long-term availability of data associated with articles in PLOS ONE

**DOI:** 10.1371/journal.pone.0272845

**Published:** 2022-08-24

**Authors:** Lisa M. Federer

**Affiliations:** Office of Strategic Initiatives, National Library of Medicine, National Institutes of Health, Bethesda, Maryland, United States of America; Tilburg University, NETHERLANDS

## Abstract

The adoption of journal policies requiring authors to include a Data Availability Statement has helped to increase the availability of research data associated with research articles. However, having a Data Availability Statement is not a guarantee that readers will be able to locate the data; even if provided with an identifier like a uniform resource locator (URL) or a digital object identifier (DOI), the data may become unavailable due to link rot and content drift. To explore the long-term availability of resources including data, code, and other digital research objects associated with papers, this study extracted 8,503 URLs and DOIs from a corpus of nearly 50,000 Data Availability Statements from papers published in PLOS ONE between 2014 and 2016. These URLs and DOIs were used to attempt to retrieve the data through both automated and manual means. Overall, 80% of the resources could be retrieved automatically, compared to much lower retrieval rates of 10–40% found in previous papers that relied on contacting authors to locate data. Because a URL or DOI might be valid but still not point to the resource, a subset of 350 URLs and 350 DOIs were manually tested, with 78% and 98% of resources, respectively, successfully retrieved. Having a DOI and being shared in a repository were both positively associated with availability. Although resources associated with older papers were slightly less likely to be available, this difference was not statistically significant, suggesting that URLs and DOIs may be an effective means for accessing data over time. These findings point to the value of including URLs and DOIs in Data Availability Statements to ensure access to data on a long-term basis.

## Introduction

In March 2014, PLOS became one of the first major journal publishers to enact a policy requiring authors to make the data underlying their publications publicly available and to include a Data Availability Statement to enable readers to locate the data [1–3]. The policy’s goal was to “facilitate data availability and transparency,” which in turn “allows validation, replication, reanalysis, new analysis, reinterpretation, or inclusion into meta-analyses, and facilitates reproducibility of research” [3]. Many publishers have subsequently developed similar policies requiring authors to make data fully and freely available at the time of publication, except where not feasible for legal or ethical reasons [4]. The International Committee of Medical Journal Editors (ICMJE) considers data sharing part of “fulfilling our ethical obligation to [clinical trial] participants” and began requiring data sharing statements in its member publications in 2018 [5]. Funders around the world have also begun requiring researchers to make their data publicly available, including, the Wellcome Trust UK Research and Innovation, the European Organization for Nuclear Research (CERN), the Japan Science and Technology Agency, the National Science Foundation (NSF), and the National Institutes of Health (NIH), which has announced a policy that will go into effect in January 2023 [6–11].

In the eight years since PLOS’s data availability policy went into effect, several studies have considered the impact of Data Availability Statements and data sharing, both in PLOS and other journals that have adopted similar policies. While the PLOS policy and others highly encourage the use of repositories as a method for sharing data, most statements do not describe sharing data in a repository, with several studies finding that only around 20% of papers have associated data in a repository [12–17]. However, even if the majority of Data Availability Statements have not achieved the ideal of sharing in a repository, studies have demonstrated that such policies do increase not only the availability of data, but also the usefulness of that data in reproducing the original study results. For example, Hardwicke et al. found an increase in the number of studies in the journal *Cognition* with data that appeared to be reusable from 22% before the journal introduced a sharing policy to 62% after the introduction of the policy, which required authors to include a Data Availability Statement [13].

While the inclusion of a Data Availability Statement does appear to help increase the likelihood that the data will be findable, these policies may require additional time on the part of authors and reviewers, with studies showing that authors and editors report spending on average five to 20 minutes more time handling papers with Data Availability Statements [18, 19]. In both studies, variations in extra time are attributed to the type of sharing described in the Data Availability Statement, although the two studies found conflicting results on which types of sharing added the most time, with the Hrynaszkiewicz and Grant study finding that statements describing sharing in a repository added the most time and Holt et al finding that statements that note the data is in the paper added the most time. Possibly because of the extra time required, as well as other perceived disadvantages of sharing data, some researchers have reported that they would avoid publishing in journals that require data sharing [20]. However, studies of citation patterns in articles with a Data Availability Statement also demonstrate that articles with statements that point to data deposited in a repository have up to 25% higher citation impact, so the extra time and effort associated with sharing data may yield benefit in the form of higher citation rates [21].

Requiring data sharing is an important step toward enhancing research reproducibility and enabling new discoveries through new analysis and meta-analysis, and the inclusion of a Data Availability Statement with research articles can significantly expedite a reader’s ability to locate the data. Previous research has shown that attempts to retrieve data made available from the author upon request are frequently unsuccessful, with only 10%-44% of requests actually yielding the relevant data [22–24]. This problem is exacerbated over time, as corresponding authors move to new institutions, change email addresses, or simply lose track of their data; Vines et al. found that the ability to successfully request and receive data fell by 17% per year after publication [24]. Stodden et al. tried contacting 180 authors who had published in *Science*, which has a policy that requires sharing data and code upon request, and only 36% of them agreed to share their data and code, with several authors expressing surprise at receiving such a request [23]. By eliminating the need to try to contact the author and simply being able to retrieve the data directly, Data Availability Statements could help ensure greater availability of data over time and serve as a useful tool for contributing to the FAIRness of data–the extent to which they are findable, accessible, interoperable, and reusable [25]–by improving findability and accessibility.

However, a Data Availability Statement is only useful in locating a dataset when the links to content remain current and when the statement contains adequate information to be able to identify the relevant data. Link rot–the process of hyperlinks ceasing to exist over time–and content drift–the changing of a web resource over time so that it eventually no longer contains the desired content–are significant problems in the scholarly literature that may also apply to the links contained in Data Availability Statements. Studies have shown that 20%-80% of scholarly articles suffer link rot, depending on their age and other factors [26–28].

Moving content off of the open web and into specialized archives designed to preserve web content may help mitigate some of these issues; for example, the Internet Archive’s Wayback Machine may help provide access to the content exactly as it appeared when the author accessed it, even if it changes or is removed over time [29]. Digital Object Identifiers (DOIs), accession numbers, and other types of persistent identifiers (PIDs) may also play a role in ensuring long-term access to resources including data, code, and other digital research objects mentioned in the literature [30]. The Joint Declaration of Data Citation Principles, which outlines a set of best practices for data citation standards, suggests citations include “a persistent method for identification that is machine actionable, globally unique, and widely used by a community,” and DOIs and other PIDs meet this definition [31].

To explore the extent to which the links contained within Data Availability Statements enable long-term access to the data and other digital objects underlying scientific articles, this study builds on previous work that described and characterized the contents of Data Availability Statements in PLOS ONE [17]. Universal resource locators (URLs) and DOIs were extracted from the corpus of nearly 50,000 Data Availability Statements from the original study and used to attempt to retrieve the referenced resources. Specifically, this study explores four questions:

1. To what extent do resources described in Data Availability Statements and associated with an identifier remain available and accessible over time?
2. Are resources with a DOI in the Data Availability Statement more likely to remain available and accessible than those with a URL?
3. Do Data Availability Statements and the associated papers contain adequate information to use the URL or DOI to locate the referenced resource?
4. Are resources shared in a repository more likely to remain available and accessible than those shared through some other method?

Questions 1 and 2 address link rot–is the URL or DOI still valid? Question 3 addresses content drift–if the URL or DOI is still valid, but the content has changed over time, such as because the site was redesigned or the data are dynamic and being constantly updated, does the Data Availability Statement or paper contain enough information that a reader would be able to locate the specific dataset referenced in the paper? Finally, question 4 considers whether repositories, which are often considered the gold standard for data sharing and recommended by PLOS’s policy and others, help contribute to long-term preservation compared to other methods of sharing such as self-archiving.

## Methods

This study used a corpus of 47,593 Data Availability Statements from all research articles published in PLOS ONE between March 1, 2014 (the date that the Data Availability Statement policy went into effect) and May 31, 2016, when the initial study corpus was collected. Data Availability Statements were downloaded using a custom R script and coded for the method of data sharing (such as in a repository, in the paper, or as supplementary information) using a combination of manual and automated coding. Additional detailed information about how this corpus was collected, processed, and analyzed is available in the Methods section of Federer et al. [17].

**Fig 1:**
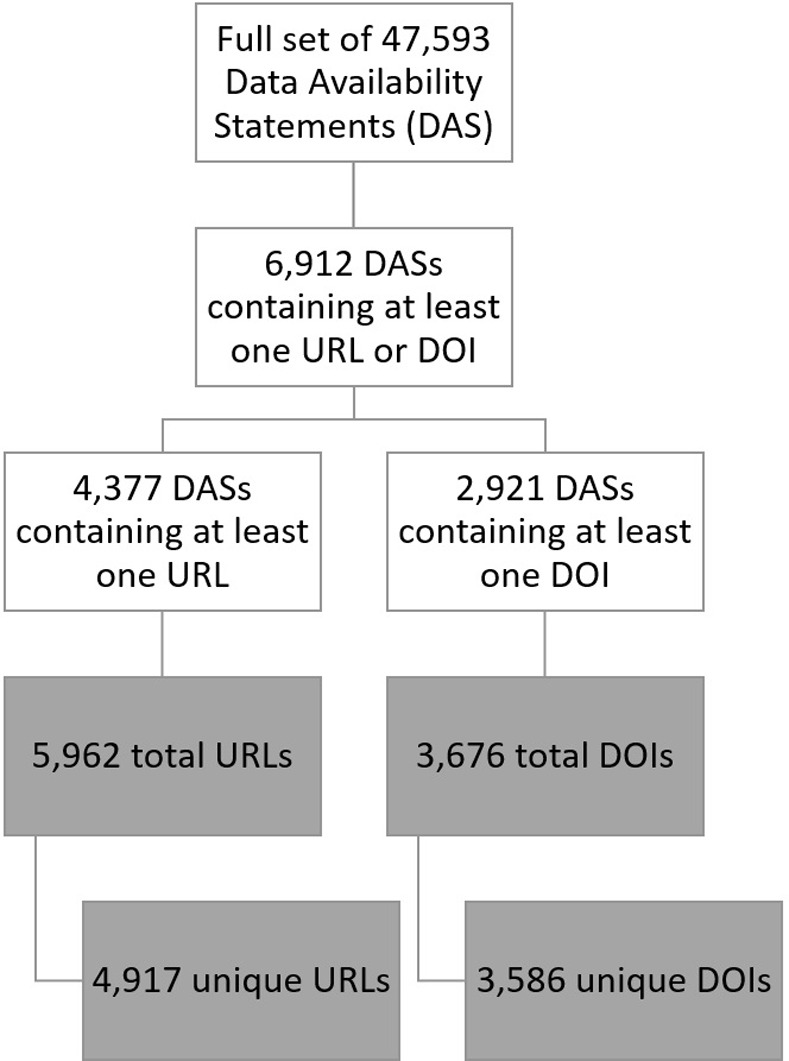
Counts of Data Availability Statements, URLs, and DOIs tested in this study.

An R script was written to extract URLs and DOIs from the Data Availability Statements by using regular expressions to identify text patterns associated with URLs and DOIs in the text. Of the 47,593 Data Availability Statements, 6,912 (14.5%) contained at least one URL or DOI, including 4,377 (9.2%) that contained at least one URL and 2,921 (6.1%) that contained at least one DOI. Some Data Availability Statements included more than one identifier, including a combination of identifiers (i.e. both a URL and a DOI), and some identifiers appeared in more than one Data Availability Statement. Accounting for duplication, this study considers 4,917 unique URLs and 3,586 unique DOIs. Fig 1 further explains the counts of Data Availability Statements, URLs, and DOIs included in the study.

Once the URLs and DOIs were extracted, a custom R script was used to attempt to fetch the resource and record the HTTP status code, which indicates whether the request was successfully completed [32]. The R script uses the curl package (version 4.3.2), which is a web client for R that allows for data transfer using a variety of network protocols [33]. The HTTP status code that the R script returns will be identical to what a human user would receive if they tried to access the URL or DOI manually via a web browser, in most cases. In a few situations, automated retrieval might fail when human manual retrieval would not. For example, some servers use rate-limiting to cap the number of requests that a user can make within a certain period of time. While a human user manually retrieving content would not likely hit this cap, the automated approach used in this R script is capable of sending requests quickly enough that it could conceivably hit the limit if many URLs or DOIs were being retrieved from the same server. It is possible to include instructions to pause between requests in the R code, but because different servers may have different limits (or no limits at all), and because most of the servers in this study were associated with only one or a small number of datasets, speed of retrieval was prioritized and no pause between requests was added. Requests that exceed the cap of allowable calls to the server will have an HTTP code of 429, so it is apparent that the failure to retrieve the content is due to rate limiting and not related to the availability of the content itself.

In a few other cases, server configurations may not support automated access or may send data in a format not supported by curl. In these cases, an error message such as “Failure when receiving data from the peer” will be returned rather than an HTTP status code, making it apparent that the failure to retrieve the content is due to the automated nature of the request and not necessarily indicative of the content being truly unavailable. If an error message was returned instead of an HTTP status code, the error message was recorded. For ease of analysis, error messages were shortened and grouped into categories; for example, the error message “schannel: next InitializeSecurityContext failed: SEC_E_CERT_EXPIRED (0x80090328)—The received certificate has expired” was coded as “sec cert expired.” For the small number of requests (n = 74, 0.7%) that returned an error message that suggested the failure to retrieve the resource might have been due to the automated nature of the request, theURLs and DOIs were checked manually to determine whether the resource could be retrieved; 59 were successfully retrieved manually. Finally, all URLs and DOIs were coded as available, not available, or manually available.

The initial availability check for the URLs and DOIs was conducted on June 8 and 9, 2021. All URLs and DOIs that were coded as not available on the first check were subsequently checked again on June 16, 2021 in case the original failure to retrieve the resource was due to a transient outage. Of the 2,030 resources that could not be retrieved on the first attempt, 183 (9%) were retrieved on the second attempt. All 183 resources retrieved on the second attempt had an HTTP status code of 429 on the first attempt, indicating too many requests had been made to the server within a specific period; all of them were either on Github or Zenodo, which were highly represented domains within this dataset.

As noted above, some identifiers appeared in more than one Data Availability Statement. Review of the most commonly appearing URLs (see Table 1) revealed that some Data Availability Statements pointed to the home page of the repository or resource containing a dataset, not the actual dataset itself. For example, many Data Availability Statements that pointed to data in the National Center for Biotechnology Information (NCBI) Gene Expression Omnibus (GEO) included the URL to the GEO homepage, along with an accession number in the Data Availability Statement that the reader could use to retrieve the data. This finding revealed a limitation of the automated approach to checking resource availability: a URL or DOI might incorrectly be recorded as available because it pointed to a high-level domain, even though the actual dataset might not be available. Additionally, the phenomenon of content drift could also lead to false positives if the URL or DOI was still active, but the content had been deaccessioned, moved elsewhere, or changed from when the author first accessed or uploaded it.

**Table 1:** Most frequently appearing URLs with resource name and count.

URL	Resource Name	Count
http://www.ncbi.nlm.nih.gov/geo	Genome Expression Omnibus (GEO)	130
http://www.ncbi.nlm.nih.gov/sra	Sequence Read Archive (SRA)	58
http://www.ncbi.nlm.nih.gov	National Center for Biotechnology Information (NCBI)	37
http://www.ncbi.nlm.nih.gov/genbank	GenBank	29
http://datadryad.org	Dryad	28
http://nhird.nhri.org.tw	National Health Insurance Research Database	27
http://proteomecentral.proteomexchange.org	Proteome Exchange	21
http://www.ncbi.nlm.nih.gov/traces/sra	Sequence Read Archive (SRA)	20

To address this limitation and test whether active URLs and DOIs coded as available actually pointed to the data, a subset of Data Availability Statements were selected for manual verification of data availability. Out of 4,430 URLs and 3,301 DOIs marked as available in the automated process, 350 URLs and 350 DOIs were randomly selected for manual verification, for a 4.9% margin of error at 95% confidence level. These URLs and DOIs were checked manually using the Chrome web browser between June 11 and June 17, 2021 and coded as either available or not available. For non-English-language web pages, the text was translated using the Google Translate function integrated into Chrome. If the page could not be translated or if the automated translation was not adequate to determine whether the page contained the resource, the URL or DOI availability was marked as NA (not applicable). Some URLs and DOIs pointed to background information rather than actual resources, such as licensing information or the homepage of a group involved in the work. These informational pages were also marked as NA.

Many URLs and DOIs pointed directly to the desired resource, but on some pages, the specific location of the actual resource was not immediately apparent; for these pages, additional information was sought from the Data Availability Statement and, if necessary, from the text of the paper itself, to attempt to locate the resource on the site. For resources that were successfully retrieved, the method of access was recorded (for example, a direct link to a resource or an accession number to search for in a larger repository). For resources that could not be retrieved with the given URL or DOI, the reason was recorded (for example, requires authorization to access).

All data processing and analysis was conducted in RStudio 1.3.959 with R version 4.0.2 [34, 35]. Tidyverse [36] was used for data wrangling and visualizations, and the custom function for automatically fetching the URLs/DOIs incorporated functions from curl version 4.3 [33], pbapply version 1.4–3 [37], and tryCatchLog version 1.2.4 [38]. Chi square tests were used to evaluate independence between groups (such as between papers of different ages or papers with a DOI versus a URL), with all tests conducted in R. All code used in this study is available on the project’s Open Science Framework repository [39].

## Results

### Availability over time

Overall, 7,790 resources could be automatically retrieved (80.8%) and 1,847 resources were unavailable (19.2%). Table 2 shows availability of resources by age of the associated paper.

**Table 2:** Availability of resources by age of the associated paper.

Age of paper	Count of resources available	Count of resources unavailable	Percent of resources available
5 years	1995	427	82.4%
6 years	4481	1095	80.4%
7 years	1314	325	80.2%

Comparing resource availability for papers in each year of age shows that resources associated with older papers are slightly less likely to be available than those associated with newer papers; however, this difference was not statistically significant (p = 0.08, χ² = 4.95, df = 2).

### Availability of URLs versus DOIs

**Fig 2:**
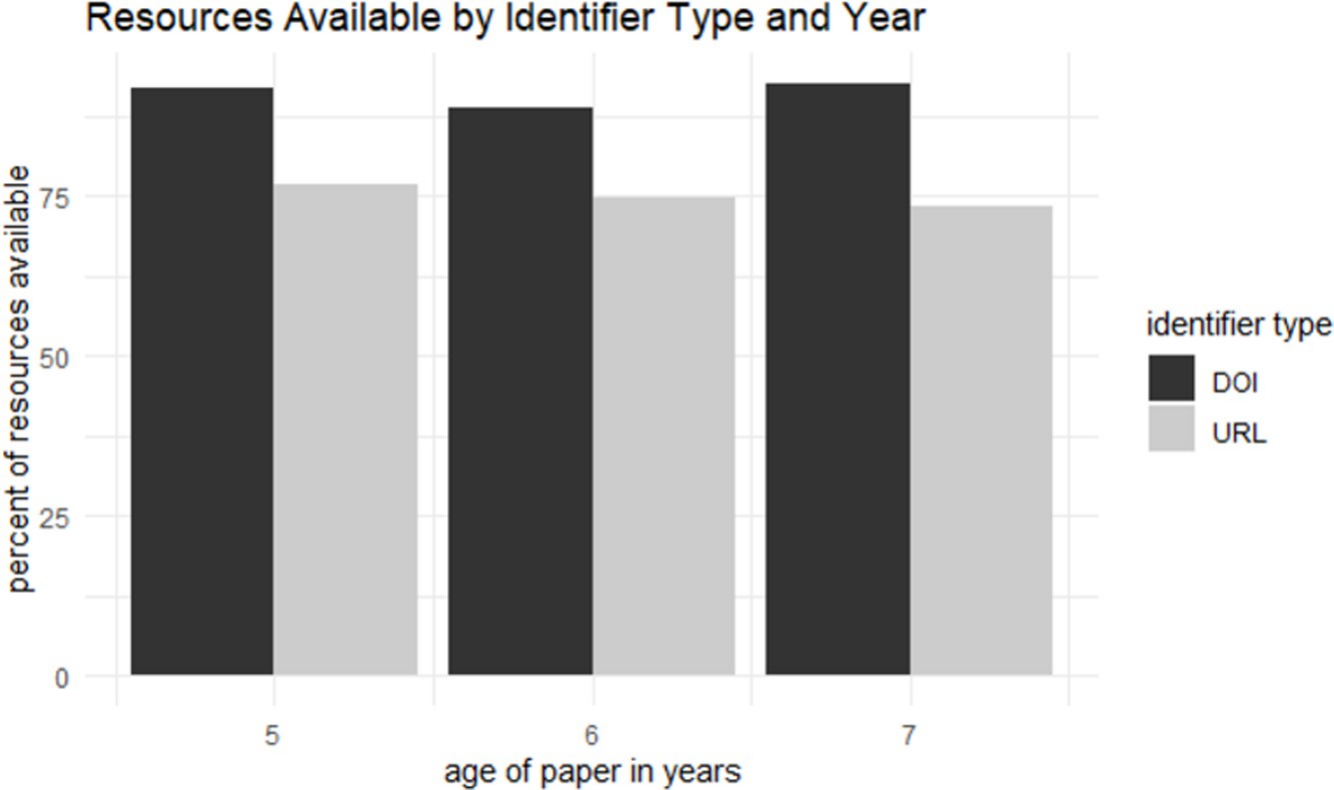
Resource availability by year and identifier type.

Using the 5,961 URLs (including duplicates), 4,474 resources (75.1%) were successfully automatically retrieved and using the 3,676 DOIs (including duplicates), 3,316 resources (90.1%) were successfully automatically retrieved. Resources with DOIs were significantly more likely to be available than URLs (p < 0.001, χ² = 335.99, df = 1). Fig 2 shows the percent of resources available through automated retrieval by age of paper and identifier type. For the URLs, the difference in availability over time was not statistically significant (p = 0.1, χ² = 4.02, df = 2). For the DOIs, there was a statistically significant difference in availability over time (p < 0.002, χ² = 12.3, df = 2), though, surprisingly, the oldest DOIs were the most likely to be retrieved.

### Ability to locate resource using Data Availability Statement

Even though a URL or DOI might be working, the page may not contain the resource; a Data Availability Statement may point only to a high-level domain, requiring the reader to locate the resource, or a resource may have moved due to content drift. To verify the actual availability of the resource, a random sample of 350 URLs and 350 DOIs were selected for manual retrieval.

Of the 350 URLs, 274 (78.2%) were located manually and 61 (22.2%) resources could not be found using the URL and the information contained within the Data Availability Statement and the paper. An additional nine resources could not be located with the URL alone, but the Data Availability Statement also contained a DOI that enabled retrieval, and six resources were coded “NA” because either the page could not be translated adequately or because the page pointed not to an actual resource, but some sort of informational page (such as a page describing licensing terms). Of the 350 DOIs, 343 (98%) were located manually, and 6 (1.7%) could not be located. One resource was coded “NA” because it pointed to an informational page. Resources with DOIs were significantly more likely to be located than those with URLs (p < 0.001, χ² = 61.8, df = 2). Considering both URLs and DOIs, 88% of resources overall (n = 617) could be located manually.

In addition to verifying whether the resource was available, the method of accessing the resource using the URL or DOI was recorded. Some URLs and DOIs pointed directly to the resource, while others require the reader to refer to the Data Availability Statement for an accession number or search the site for the data using information from the Data Availability Statement. Because PLOS guidelines do allow authors to restrict access to sensitive data, some URLs pointed not to the actual resource, but to instructions about how to request the data. Table 3 shows access methods for URLs and DOIs. Besides being more likely to be able to be retrieved, DOIs were almost universally direct links, which make it easier and quicker for the reader to locate the dataset than having to search the site for the data or request it.

**Table 3:** Method of access for resources by identifier type for manually retrieved resources.

Access Method	URLs (274 total)		DOIs (343 total)	
	N	%	n	%
Direct link	169	61.7%	341	99.4%
Accession number	43	15.7%	0	0%
Search	33	12%	0	0%
Instructions for requests	29	10.6%	2	0.6%

For each resource that could not be retrieved, the reason for being unable to locate it was recorded. Among the 61 URLs for which the resource could not be retrieved, the majority of the resources (43, 70.5%) could not be located because the Data Availability Statement and the paper lacked adequate information to find it on the site. For example, one Data Availability Statement stated “the data used in this paper is publically available through [name redacted] university’s research repository ([url redacted]),” but neither the Data Availability Statement nor the paper provided details about the specific location within the repository or the name of the dataset, nor did searching for the author’s name enable locating the data. Six URLs pointed to sites that required authorization to access, four produced “page not found” errors, and two had server/database errors. Two pointed to sites that had been restructured and did not include forwarding to the resource’s new location, two pointed to sites that were out of date and the links to the resource no longer worked, one pointed to a site that noted the data had been deaccessioned, and one Data Availability Statement contained an incorrect accession number. Among the six DOIs for which the resource could not be retrieved, three pointed to pages that required authorization to access, two were unable to be located due to inadequate information in the Data Availability Statement or the paper, and one DOI resolved to an incorrect page.

### Availability of resources by sharing type

In the original analysis of the Data Availability Statement corpus, each statement was coded with the method used for data sharing, such as upon request, in the paper or supplemental information, or in a repository. About 20% of the Data Availability Statements in the corpus report sharing their data in a repository; of the 9,637 URLs and DOIs in the subset of Data Availability Statements used for this analysis, 6,908 (71.7%) were associated with a repository. This finding is logical, given that Data Availability Statements reporting the data being shared in the paper itself or its supplemental information would typically not need to report a URL or DOI. Resources shared in a repository were significantly more likely to be available than those shared through some other method (p < 0.001, χ² = 270.36, df = 8), with 84.3% of resources shared in a repository remaining available compared to 72% shared through some other method. Resources shared in a repository were also significantly more likely to be associated with a DOI than a URL (p < 0.001, χ² = 842.3, df = 8); 46.3% of resources in a repository were associated with a DOI compared to 17.6% for other types of sharing.

## Discussion

This study considered Data Availability Statements associated with articles published in PLOS ONE between March 1, 2014 (the date that the Data Availability Statement policy went into effect) and May 31, 2016 to evaluate the long-term availability of resources associated with URLs and DOIs. In summary, the findings of this study have answered the following research questions:

1. To what extent do resources described in Data Availability Statements and associated with an identifier remain available and accessible over time?
The majority of resources (more than 80%) associated with a URL or DOI in a Data Availability Statement in this sample remain available and accessible.
2. Are resources with a DOI in the Data Availability Statement more likely to remain available and accessible than those with only a URL?
Resources associated with DOIs are significantly more likely to be available than those associated with URLs.
3. Do Data Availability Statements and the associated papers contain adequate information to use the URL or DOI to locate the specific dataset or resource referenced by the article?
The majority of resources (88%) could be located using the information contained within the Data Availability Statement. Resources associated with DOIs were significantly more likely to be findable than those associated with URLs.
4.Are resources shared in a repository more likely to remain available and accessible than those shared through some other method?
Most resources in this study were shared in a repository, and those that were shared through some other means were significantly less likely to be available than those shared in a repository.

This study has demonstrated that Data Availability Statements with URLs and DOIs make the data and code associated with papers more likely to be available over time. Previous studies have demonstrated that obtaining data and code upon request is often fruitless, with some authors unable to be reached and others being unwilling to share their data or unable to do so because it is no longer available [22–24]. For example, Vines et al.’s study, which attempted to obtain data by requesting from the author, found only 26% of data extant with six-year-old papers compared with 80% of six-year-old papers in this study [24]. In other words, resources in this study with URLs and DOIs were nearly 12 times more likely to be available than those of the same age that had to be requested in the Vines et al. study. Resources associated with DOIs were even more likely to be available; resources associated with six-year-old papers with URLs were seven times more likely to be available than requested articles of the same age in Vines et al., while resources with DOIs were 23 times more likely to be available.

However, it should also be noted that disciplinary differences may contribute to variations in rates of data availability between the present study and Vines et al.’s study. While this study considers papers from *PLOS ONE*, which covers a wide range of subjects, the paper in Vines et al.’s study covers not only a specific discipline (biology) but also a very specific data type (“morphological data from plants or animals that made use of a discriminant function analysis”) [24]. Previous studies have found disciplinary differences in researchers’ sharing practices, which could contribute to differences in data availability when comparing papers across disciplines (although, interestingly, in Tenopir et al.’s studies of data sharing, which were published around the same time as Vines et al.’s study, biologists were among the most likely to believe that others could access their data easily) [40, 41]. Future research could explore whether disciplinary differences in data sharing also translate to differences in the rates at which data remain available over time.

Data Availability Statements that contain a direct link to the associated data make it easy for any reader to access the data without having to rely upon the author. Data Availability Statements also lessen the burden on authors who would have otherwise received such requests and had to locate, prepare, and share their data. By making data easy to locate, Data Availability Statements help enhance the FAIRness of data, increasing findability and accessibility.

However, this study has also demonstrated that Data Availability Statements are not a panacea for data sharing. Although the resources in this study were far more likely to be available than resources in previous studies that required contacting the author, not all Data Availability Statements successfully lead the reader to the resource. URLs in particular are susceptible to link rot and content drift; authors should consider how to ensure long-term viability of the data by ensuring that the URL in the Data Availability Statement will be maintained over time. Placing the data in a recognized repository may be a useful solution for ensuring this long-term availability and is also “strongly recommended” by the PLOS Data Availability policy [42]. Using a repository takes the burden of ongoing maintenance of the data off the author and may make data less susceptible to link rot. Data in a repository is also more likely to have a DOI, which is also associated with greater availability.

Obtaining a DOI may also be desirable for datasets that are not placed in a repository, though DOIs do require some maintenance to ensure that they are updated if the resource moves. DOIs in this study were more likely to be direct links, which can make it easier for readers to easily locate the dataset as opposed to having to search the website. DOIs also have the benefit of being more easily tracked and used for metrics to quantify data reuse, with initiatives like SCHOLIX and Make Data Count aimed at enhancing the infrastructure for tracking DOIs and understanding how to use and interpret data metrics [43, 44]. Some academic libraries have become members of DataCite, the primary service for providing PIDs for data and other digital research objects, to provide DOI minting services for their institutions.

This study does have some limitations that could be addressed in future research. Because PLOS only began requiring Data Availability Statements in mid-2014, this study is necessarily limited in the age of papers that can be considered. The oldest papers in this study were seven years old, which is comparatively young. This study also considered Data Availability Statements only up to mid-2016, since that was the period of collection for the initial study in which this dataset was collected. Although availability did not differ significantly over time in this study, patterns of availability may differ for newer papers. As time goes on, revisiting this study may provide additional insight into longer-term availability. This study considered Data Availability Statements only from PLOS ONE; future analysis could include other journals or publishers to determine whether the patterns of availability seen here hold true across the scientific literature in general.

In addition, this study used an automated approach to retrieve URLs and DOIs, which has the potential to either overestimate or underestimate true availability of resources, depending on several factors. False positives may result when a page still exists at the URL, but it no longer contains the dataset referenced in the Data Availability Statement. In the subset of URLs in this study that were retrieved manually, about 20% were determined to be false positives, so the actual rate of available resources may be lower than the automated approach suggests. False negatives are also possible, though less likely, since HTTP codes and error messages can be used to determine whether failure to retrieve the resource was due to the automated nature of the request, such as in the case of HTTP 429 codes that indicate the resource could not be retrieved because the rate limit was exceeded. Augmenting this automated approach with manual checking of URLs and DOIs can help determine whether the dataset is available at the stated location, but does not enable a determination of whether the dataset is accurate, complete, or, as Stodden et al. considered, sufficient for reproducing the findings of the paper [23]. Such investigation would require significant subject matter expertise in the science behind the dataset, and, given the broad scope of subjects covered in PLOS ONE, would likely need to be limited to a smaller subset of studies for which the study team had adequate background knowledge.

## Conclusion

As more journals and funders begin requiring data sharing, it is useful to understand best practices for ensuring that data are FAIR. Mandating the inclusion of a Data Availability Statement with papers can be helpful in ensuring that readers can easily locate the resources associated with a study not only upon its publication but for the longer term. As this study has demonstrated, in comparison with previous studies that attempted to obtain data by contacting the author, including a URL or DOI in the Data Availability Statement could make data significantly more likely to be retrieved than if the reader must rely on the author to provide the data.

These findings may have implications that journals and funders could consider when creating policy around data sharing to ensure the maximum benefit to readers and to the broader scientific community. Simply requiring that data be shared in some form may not have the desired impact of making scientific data FAIR, as studies have repeatedly demonstrated that many datasets that are ostensibly shared may not actually be accessible. Ensuring that data are truly available, and remain so over the long term, may necessitate additional requirements and specifications about how and where data are shared. Requiring that researchers include a URL or DOI in documentation such as Data Availability Statements (for journals) or progress reports (for funders) may help increase the extent to which data are FAIR and accessible on a long-term basis.
